# World-wide variation in incidence of *Acinetobacter* associated ventilator associated pneumonia: a meta-regression

**DOI:** 10.1186/s12879-016-1921-4

**Published:** 2016-10-18

**Authors:** James C. Hurley

**Affiliations:** 1 Department of Rural Health, Melbourne Medical School, University of Melbourne, Ballarat, 3353 Australia; 2 Internal Medicine Service, Ballarat Health Services, PO Box 577, Ballarat, 3353 Australia; 3 Infection Control Committees, St John of God Hospital and Ballarat Health Services, Ballarat, Victoria Australia

**Keywords:** Intensive care unit, Geographic variation, Ventilator associated pneumonia, *Acinetobacter*

## Abstract

**Background:**

*Acinetobacter* species such as *Acinetobacter baumanii* are of increasing concern in association with ventilator associated pneumonia (VAP). In the ICU, *Acinetobacter* infections are known to be subject to seasonal variation but the extent of geographic variation is unclear. The objective here is to define the extent and possible reasons for geographic variation for *Acinetobacter* associated VAP whether or not these isolates are reported as *Acinetobacter baumanii*.

**Methods:**

A meta-regression model of VAP associated *Acinetobacter* incidence within the published literature was undertaken using random effects methods. This model incorporated group level factors such as proportion of trauma admissions, year of publication and reporting practices for Acinetobacter infection.

**Results:**

The search identified 117 studies from seven worldwide regions over 29 years. There is significant variation in *Acinetobacter* species associated VAP incidence among seven world-wide regions. The highest incidence is amongst reports from the Middle East (mean; 95 % confidence interval; 8.8; 6 · 2–12 · 7 per 1000 mechanical ventilation days) versus that from North American ICU’s (1 · 2; 0 · 8–2 · 1). There is a similar geographic related disparity in incidence among studies reporting specifically as *Acinetobacter baumanii*. The incidence in ICU’s with a majority of admission being for trauma is >2.5 times that of other ICU’s.

**Conclusion:**

There is greater than fivefold variation in *Acinetobacter* associated VAP among reports from various geographic regions worldwide. This variation is not explainable by variations in rates of VAP overall, admissions for trauma, publication year or Acinetobacter reporting practices as group level variables.

**Electronic supplementary material:**

The online version of this article (doi:10.1186/s12879-016-1921-4) contains supplementary material, which is available to authorized users.

## Background

*Acinetobacter* species are opportunistic gram negative bacteria which are of emerging concern in intensive care units worldwide [1–102]. *Acinetobacter* species accounted for 7.9 % of bronchoscopically documented ventilator associated pneumonia in a series drawn predominantly from centres in Europe and The United States of America [103]. In contrast to other VAP pathogens, *Acinetobacter* species varies in incidence worldwide for reasons that remain to be fully defined.

*Acinetobacter baumannii* has the greatest clinical importance amongst *Acinetobacter* species as it is typically associated with outbreaks in the hospital setting and it has major antimicrobial resistance issues. There is evidence for [104] and against [105] an increase in attributable mortality in association with *Acinetobacter baumannii* infections in the ICU.

Any survey of the worldwide epidemiology of Acinetobacter associated with VAP would be challenging for several reasons. First is that Acinetobacter infections in ICU are subject to seasonal influences [106–108]. For example, this is most apparent for *Acinetobacter* species blood stream infections versus other bacterial isolates such as *Pseudomonas aeruginosa* and appears to correspond with the higher outdoor air temperature [107]. For example, for each 10 °F increase, Perencevich et al. observed a 17 % increase in the monthly rates of infection from multiple body sites caused by *A. baumanii* at the University of Maryland Medical Center over a seven year period [108].

The second challenge is its potentially complex epidemiology. *A. baumanii* infection has the potential for the simultaneous occurrence of endogenous outbreak and non-outbreak strains in an ICU together with, in tropical areas, natural disasters and military deployments, occasional community acquired infections [70, 109–111].

However, the greatest difficulty is in the vagaries and variations in the reporting practices of Acinetobacter species. The differentiation among Acinetobacter species may be challenging for a busy clinical microbiology laboratory as the microbiological identification on the basis of phenotypic characteristics is difficult [101, 102]. As a consequence, the reporting and documentation to Acinetobacter species level may not be uniform across laboratories around the world and may be a high priority only in low incidence countries. While *Acinetobacter baumannii* is reported as the most frequently isolated species (>90 percent of Acinetobacter species isolates) [101], this predominance may reflect the reporting practices of clinical microbiology laboratories.

Regional variation in the worldwide incidence has previously been described [102]. However, the quantification of this variation and moreover, the degree to which it may be explainable, are uncertain. The objective here is to define the extent of geographic variation within the published literature using meta-regression methods.

## Methods

The literature search and analytic approach used here is as described previously [112]. In brief, an electronic search of PubMed, The Cochrane database and Google Scholar for systematic reviews containing potentially eligible studies was undertaken using the following search terms; “ventilator associated pneumonia”, “mechanical ventilation”, “intensive care unit”, up to June 2016. This search was expanded to include reports that used the number of mechanical ventilation days as the denominator in addition to those reports that used the number of patients receiving prolonged mechanical ventilation as the denominator. These publications were reviewed for listing of VAP isolates including *Acinetobacter.* Because this analysis was based on a literature survey, institutional review board approval was not required.

The VAP associated *Acinetobacter* is the number of patients with VAP having an *Acinetobacter* species isolated from respiratory sampling. Where necessary, this number was derived as the number of patients with VAP multiplied by the proportion of VAP isolates that were *Acinetobacter* species. In addition, the following were also extracted where available; the number of ICU patients surveyed, the overall incidence of VAP per 1000 mechanically ventilated day (MVD), whether the mode of diagnosis of VAP required bronchoscopic sampling and whether the ICU was a trauma ICU (defined as more than 50 % of admissions for trauma). Also, whether the mode of reporting of Acinetobacter infection was as *Acinetobacter baumanii* versus other modes such as *Acinetobacter species* was used as an indicator variable.

The assignment of countries to near neighbour groupings was solely determined in relation to geographic proximity without regard to political, economic or other considerations. For the purpose of generating a world map of Acinetobacter VAP incidence by country, summary rates by country were estimated were at least two study reports were available for that country.

A meta-regression model of VAP associated *Acinetobacter* was undertaken. The weight in this model is the inverse of the study variance. Because heterogeneity both within and between regions is to be expected, a random effects method was used for these estimates. The predictor variables in the regression model were the region from where the study originated, the mode of diagnosis of VAP, mode of reporting of Acinetobacter infection, trauma ICU and year of publication.

## Results

The search identified 117 studies contained in 100 publications (Additional file 1) published over a period spanning 29 years [1–100]. The studies are detailed in the Additional file 1: Table S1-S6. The studies were classified by geographic region as detailed in Table 1. There were 13 multinational ICU surveys from four publications which were classified separately (Additional file 1: Tables S1) as the incidence data in each of these ICU’s were anonymized by originating country in these publications. The majority of the ICU’s in these multinational studies were from outside of Europe and North America.

**Table 1:** Characteristics of studies^a^

	Multinational	Europe^b^	Mediterranean^b^	Asia^b^	Middle East^b^	Central & South America^b^	USA/Canada^b^	Ungrouped^b^
Sources [ref]	Additional file 1: Table S1 [S1-S4]	Additional file 1: Table S2 [S5-S37]	Additional file 1: Table S3 [S38-S57]	Additional file 1: Table S4 [S58-S64]	Additional file 1: Table S4 [S65-S73]	Additional file 1: Table S5 [S74-S81]	Additional file 1: Table S5 [S82-S96]	Additional file 1: Table S6 [S97-S100]
Number of groups	13	35	20	7	11	9	18	4
MV for >48 h for <75 %^c^	0	1	1	1	0	1	2	0
Trauma ICUs^d^	1	2	6	0	4	0	6	0
Bronchoscopic sampling^e^	3	18	13	1	1	1	7	1
Intervention period	1	2	0	0	1	1	1	0
Study publication year (range)	1993-2012	1988-2016	1987-2012	2001-2016	2000-2013	1995-2013	1987-2014	1987-2015
Numbers of patients per study group; median (IQR)	2082; 1029-3413	385; 145-764	194; 101-318	952; 301-16426	260; 100-724	270; 233-427	340; 277-678	174; 65-331
Duration of MV (days); median (IQR)	6; 5-7	11; 7.4-14	8; 6.5-10.2	7.5; 6-9	8.9; 7.1-10	9.6; 7.6-10	5.5; 4–10.5	9.2; 4–10.6
VAP incidence;								
• per 1000 ventilator days; o mean; ^f^ o 95 % CI	30 · 6; 20 · 4–40 · 7	24 · 3; 18 · 1–30 · 4	29.8 % 21 · 4–38 · 2	29 · 7; 15.9–43 · 5	34 · 0; 22.9–44 · 9	31 · 5; 19 · 3–43 · 6	26 · 7; 17 · 9–35 · 5	33 · 7; 15 · 4–51 · 9
*Acinetobacter* (all) VAP incidence^g^								
• per 1000 ventilator days; o mean; ^h^ o 95 % CI	4 · 2; 2 · 8–6 · 2	1 · 3; 0.7–2 · 5	3 · 5; 2.0–6 · 1	5.5; 2 · 4–12 · 8	8.8; 6 · 2–12 · 7	3 · 3; 1 · 8–6 · 2	1 · 2; 0 · 8–2 · 1	3 · 1; 1 · 9–5 · 2
*Acinetobacter baumanii* VAP incidence ^i^								
• per 1000 ventilator days; o mean; ^j^ o 95 % CI	8 · 2; 0 · 7–3 · 5	0 · 51; 0.1–0 · 9	2 · 8; 1 · 4–5 · 4	6.6; 2 · 1–20 · 1	18.0; 9 · 8–33 · 1	4 · 4; 2 · 0–10 · 0	1 · 2; 0 · 6–2 · 6	

^a^Abbreviations; ICU, Intensive care unit; MV; Mechanical ventilation; NA not applicable; VAP ventilator associated pneumonia; IQR, interquartile range

^b^Europe includes France, Germany, United Kingdom, Switzerland, Sweden, Iceland, and Poland; Mediterranean includes Spain, Italy, Greece and Tunisia; Asia includes China, India, Pakistan and Bangladesh; Middle East includes Turkey, Iraq, Lebanon and Saudi Arabia; Central & South America includes Argentina, Brazil, Chile, Colombia, Cuba and Guatemala; Northern America includes USA and Canada; Ungrouped includes Australia and South Africa

^c^Studies for which less than 75 % of patients were reported to receive more than 48 h of mechanical ventilation

^d^Trauma ICU defined as an ICU with >50 % of patient admissions for trauma

^e^Bronchoscopic versus tracheal sampling toward the diagnosis of VAP

^f^Mean VAP incidence (per 1000 MV days) was not significantly different between the six geographic regions; p = 0.74

^g^Acinetobacter (all) refers to Acinetobacter regardless of whether listing in the original study was as Acinetobacter species, *Acinetobacter baumanii*, or other speciation

^h^Mean *Acinetobacter* VAP incidence (per 1000 MV days) was significantly different between the six geographic regions; p = 0.003

^i^Only from those studies that specified *Acinetobacter baumanii*

^j^Mean *Acinetobacter* VAP incidence from studies reporting as *Acinetobacter baumanii* (per 1000 MV days) was significantly different between the six geographic regions; p = 0.014

While none of the studies were undertaken in the context of an outbreak, six studies were undertaken in the context of an infection control intervention targeting overall ICU infection rates generally [4, 5, 72, 75, 92] or VAP infections specifically [70]. The period of study ranged from 1 to 150 months. There were 11 studies [1, 44, 54, 60, 69, 74, 83, 86, 91, 92, 97] that could have been subject to seasonal variation in Acinetobacter incidence as the period of study in each was less than 12 months. These were excluded from the meta-regression model. There were 18 studies that reported for trauma ICU populations [2, 22, 32, 40, 43, 47, 50, 53, 55, 66, 67, 70, 82,-85, 94, 95].

The Acinetobacter associated with VAP was reported most commonly as Acinetobacter without speciation (i.e. Acinetobacter species; 53 studies). Acinetobacter VAP infections were less commonly reported as follows; *Acinetobacter baumanii* (47 studies); *Acinetobacter calcoaceticus* (four studies)*;* and *Acinetobacter anitratus* (two studies). There was no instance of any study reporting more than one Acinetobacter species type.

There was no significant difference in the overall VAP incidence across the region categories (*p* = 0.36; Table 1; Additional file 1: Table S7). There was a significant variation in mean VAP associated *Acinetobacter* across the region categories (Fig. 6; *p* = 0.003) with the *Acinetobacter* species associated VAP incidence being highest amongst reports from ICU’s in the Middle East (mean; 95 % confidence interval; 8.8; 6 · 2–12 · 7 per 1000 mechanical ventilation days) versus reports from Northern Europe (1.3; 0.7–2 · 5) and North American ICU’s (1 · 2; 0 · 8–2.1) (Table 1).

**Fig. 1:**
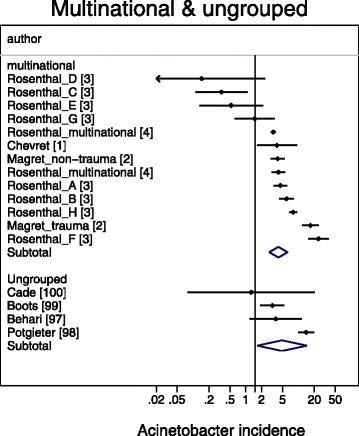
Caterpillar plots of the group specific (small diamonds) and summary (large open diamond, broken vertical line) Acinetobacter VAP incidence per 1000 mechanical ventilation days and 95 % CI for groups from the multinational (top) and ungrouped (bottom) studies. For comparison, the summary Acinetobacter VAP incidence (vertical line) derived from the studies from French groups is shown for reference. Studies are listed in Additional file 1: Tables S1 & S6. Note that the x axis is a logarithmic scale

**Fig. 2:**
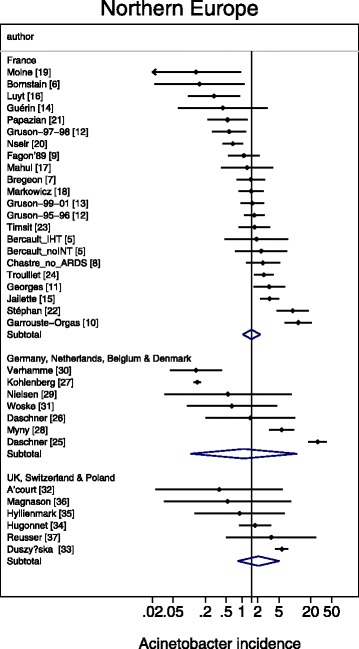
Caterpillar plots of the group specific (small diamonds) and summary (large open diamond) Acinetobacter VAP incidence per 1000 mechanical ventilation days and 95 % CI for groups from the French (top) studies, and studies from other European countries. For comparison, the summary Acinetobacter VAP incidence (vertical line) derived from the studies from French groups is shown for reference. Studies are listed in Additional file 1: Tables S2. Note that the x axis is a logarithmic scale

**Fig. 3:**
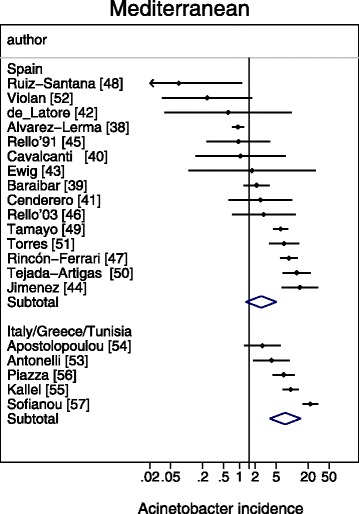
Caterpillar plots of the group specific (small diamonds) and summary (large open diamond) Acinetobacter VAP incidence per 1000 mechanical ventilation days and 95 % CI for groups from the Mediterranean studies. For comparison, the summary Acinetobacter VAP incidence (vertical line) derived from the studies from French groups is shown for reference. Studies are listed in Additional file 1: Tables S3. Note that the x axis is a logarithmic scale

**Fig. 4:**
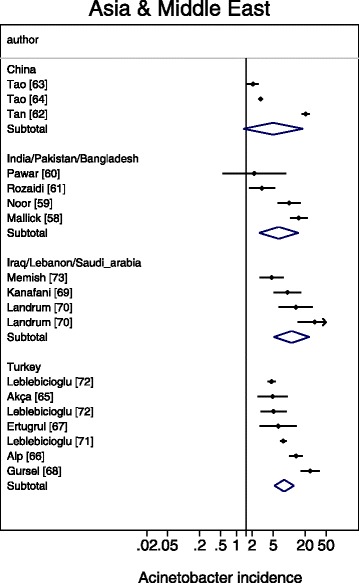
Caterpillar plots of the group specific (small diamonds) and summary (large open diamond) Acinetobacter VAP incidence per 1000 mechanical ventilation days and 95 % CI for groups from the studies from Asia (top) and the middle East (bottom). For comparison, the summary Acinetobacter VAP incidence (vertical line) derived from the studies from French groups is shown for reference. Studies are listed in Additional file 1: Tables S4. Note that the x axis is a logarithmic scale

**Fig. 5:**
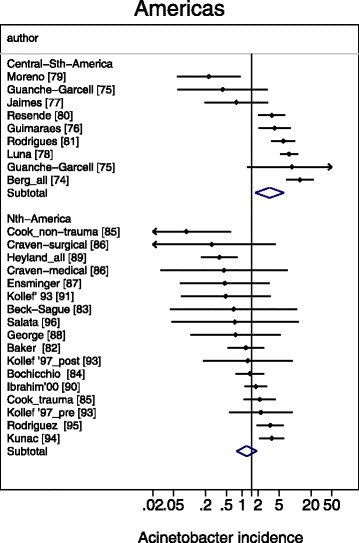
Caterpillar plots of the group specific (small diamonds) and summary (large open diamond) Acinetobacter VAP incidence per 1000 mechanical ventilation days and 95 % CI for groups from the North (bottom) and central and outh (top) American studies. For comparison, the summary Acinetobacter VAP incidence (vertical line) derived from the studies from French groups is shown for reference. Studies are listed in Additional file 1: Tables S5. Note that the x axis is a logarithmic scale

**Fig. 6:**
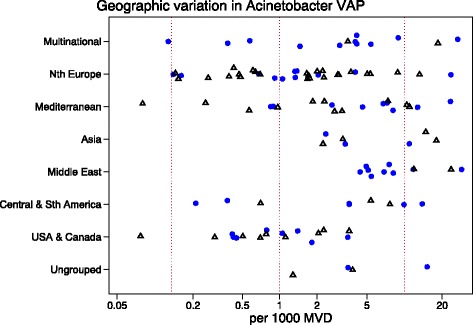
A scatter plot of worldwide *Acinetobacter* VAP incidence (per 1000 MV days) among published studies in seven geographic regions with rates from studies reporting Acinetobacter infections as *Acinetobacter baumanii* (open symbols) versus reporting as Acinetobacter species or otherwise (closed symbols; Note logarithmic scale of incidence). The vertical lines are for reference at incidence rates of 0.1, 1 and 10 per 1000 mvd

A meta-regression of Log Acinetobacter VAP incidence per thousand MV days revealed no significant association with use of bronchoscopy for VAP diagnosis, or year of publication for *Acinetobacter* species associated VAP incidence (Table 2). For the purpose of the meta-regression and also for the caterpillar plots (Figs. 1, 2, 3, 4 and 5), the incidence in French studies was used as the benchmark incidence given that the largest number of studies originated from French ICU’s. Both origin of study from the Middle East and also origin from a trauma ICU were each significant factors for a positive association for *Acinetobacter* species associated VAP incidence. Surprisingly, the mode of reporting of Acinetobacter VAP infection, whether as *Acinetobacter baumanii* versus other modes such as *Acinetobacter species*, was not a significant factor in these models. This is apparent in a summary figure for all studies (Fig. 6). The results of a meta-regression model limited to those studies that specifically reported as *Acinetobacter baumanii* gave similar findings (Table 2).

**Table 2:** Log *Acinetobacter* VAP incidence per thousand MV days; meta-regression models^a^

	Studies reporting as either Acinetobacter species or *Acinetobacter baumanii*			Only studies reporting as *Acinetobacter baumanii*		
Factor	Coefficient ^b^	95 % CI	p	Coefficient ^b^	95 % CI	p
Northern European studies (reference group)	+0 · 62	−0 · 37 - +1 · 6		+0 · 71	−2 · 43 - +1 · 01	
Geographic region						
• Mediterranean	+0 · 65	−0 · 10 - +1 · 40	0 · 09	+0 · 33	−0 · 67 - +1 · 33	0 · 51
• Asia	+0 · 71	−0 · 42 - +1 · 83	0 · 22	+0 · 05	−1 · 57 - +1 · 66	0 · 95
• Middle East	+1 · 21	+0 · 28 - +2 · 13	0 · 01	+1 · 74	+0 · 20 - +3 · 28	0 · 03
• Central & South America	+0 · 53	−0 · 64 - +1 · 70	0 · 37	+0 · 56	−1 · 05 - +2 · 18	0 · 48
• USA & Canada	−0 · 90	−1 · 76 - -0 · 04	0 · 04	−1 · 15	−2 · 45 - +0 · 15	0 · 08
• Ungrouped	+0.28	−1 · 56 - +2.10	0.77	+0.31	−3 · 36 - +3.98	0.87
• Multinational	+0.64	−0 · 22 - +1.49	0.14	+0.75	−0 · 89 - +2.4	0.36
Trauma^c^	+0 · 93	+0 · 26 - +1.59	0 · 007	+0 · 97	+0 · 001 - +1.94	0 · 05
Year of publication^d^	+0 · 02	−0 · 04 - +0 · 04	0 · 92	+0 · 05	−0 · 02 - +0 · 12	0 · 14
Mode of diagnosis^e^	−0 · 26	−0 · 81 - +0 · 28	0 · 34	+0 · 10	−0 · 71 - +0 · 91	0 · 80
Intervention period^f^	−0.31	−1.4 - +0.78	0.57	+0.22	−1.58 − +2.02	0.81
*Acinetobacter baumanii* ^g^	−0 · 06	−0 · 61 - +0 · 50	0 · 84			

^a^This table displays the results of a meta-regression analysis for log *Acinetobacter* VAP incidence per thousand MV days

^b^Interpretation. The reference group is the Northern European studies and this coefficient equals the difference in log from 0 (a log equal to 0 equates to a rate of 1. The other coefficients represent the difference in log for groups positive for that factor versus the reference group

^c^The co-efficient for trauma represents the increment in log for an ICU having a majority of admissions for trauma

^d^The co-efficient for year of publication represents the linear increment in log for each year after 1980

^e^For sampling using bronchoscopic versus tracheal sampling

^f^Studies undertaken during an infection control intervention

^g^Studies reporting Acinetobacter infections as *Acinetobacter baumanii* versus reporting as Acinetobacter species or otherwise

**Fig. 7:**
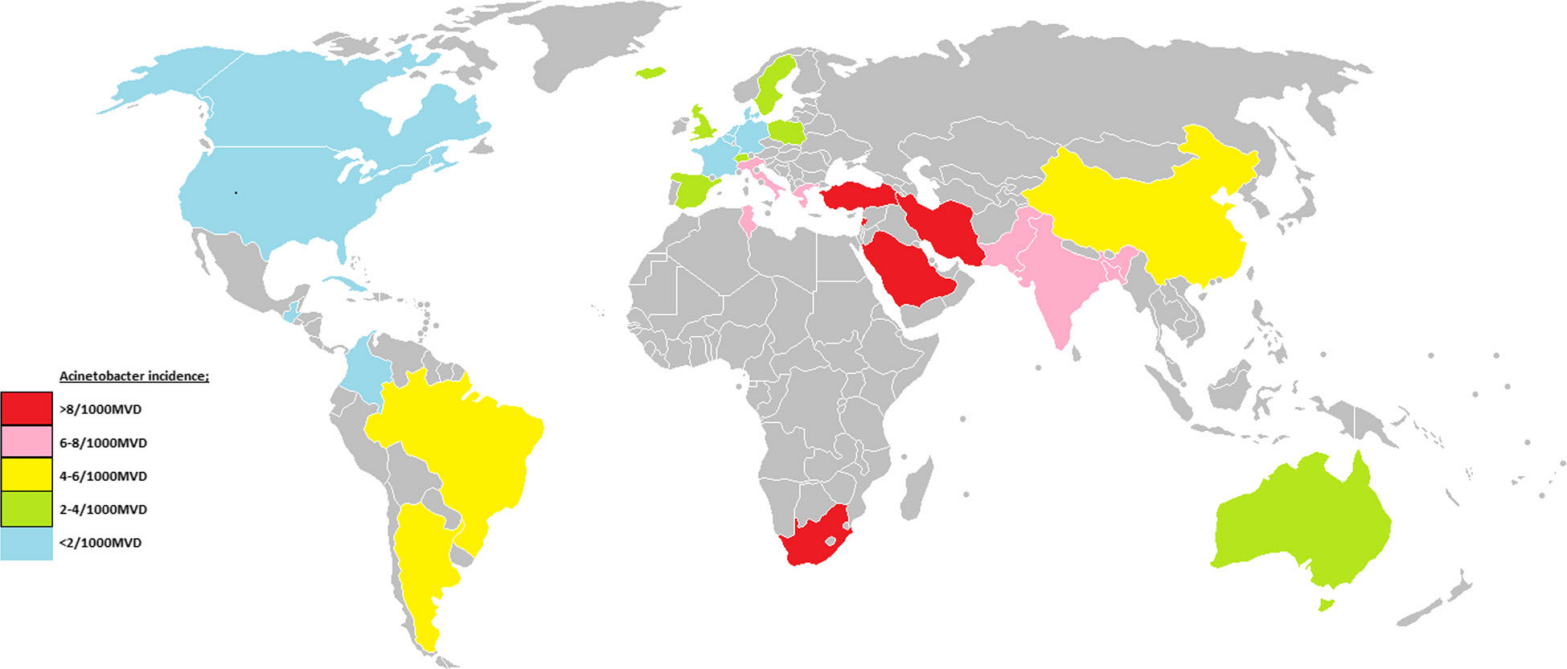
A map of *Acinetobacter* VAP incidence per 1000 MV days for each country or world sub region for which at least two reports were available. Colour code; *red* >8; *pink* 6–8; *yellow* 4–6; *green* 2–4; *blue* < 2. Map modified from the following source; https://commons.wikimedia.org/wiki/File%3ABlankMap-World-v2.png By original uploader: Roke (Own work) [GFDL (http://www.gnu.org/copyleft/fdl.html) or CC-BY-SA-3.0 (http://creativecommons.org/licenses/by-sa/3.0/)], via Wikimedia Commons from Wikimedia Commons

On the basis of the results of studies reporting incidence for single countries, a world map of Acinetobacter VAP infection incidence can be produced (Fig. 7).

## Discussion

This is a survey of the incidence of *Acinetobacter* species associated VAP among published studies using meta-analysis to characterize the variation in incidence worldwide. It reinforces and further characterizes previous observations [101, 102]. It reveals a more than fivefold variation in incidence among seven broad world-wide and multinational regions that is not explainable by a limited number of group level factors.

There are major logistical challenges in undertaking any international survey and there are few prospective multinational comparisons of hospital acquired infections published. A worldwide prevalence survey of *Pseudomonas aeruginosa* associated VAP across 11 countries during 2011–2012 revealed an insignificant variation in prevalence of both *P. aeruginosa* ventilator-associated pneumonia and also VAP overall across four regions; the United States, Europe, Latin America, and Asia Pacific [113]. By contrast, an anonymized survey of 55 ICUs of 46 hospitals in Argentina, Brazil, Colombia, India, Mexico, Morocco, Peru, and Turkey revealed an overall rate of VAP of 24.1 per 1000 MV days with *Acinetobacter* species accounting for between 3 and 46 % of VAP isolates amongst the eight non-identified countries [3]. However, the extent to which any possible association with admission for trauma account for differences in VAP microbiology is difficult to establish in short term single center studies [114, 115].

Seasonal variation is another challenge to attempts at surveillance [106–108]. The seasonal variation amongst hospital acquired pneumonia and bloodstream *Acinetobacter* species infections was first documented in National Nosocomial Infections Surveillance System (NNIS) data and more recently within The Surveillance Network-USA database [106]. *Acinetobacter* species infections in these surveys were ~50 % more common in summer than winter months. The variation seen here in this worldwide survey exceeds that explainable by seasonal variation. A possible mechanism to account for this seasonal and possibly geographic variation, and by contrast to species that do not exhibit the same variation, is that Acinetobacter and particularly *A. baumanii* have an exceptional ability to survive desiccation. It remains to speculate how this property of Acinetobacter could account for the variation found here. Of interest in this regard however, amongst a panel of Acinetobacter isolates, this ability to survive desiccation was notable for *A. baumanii* that had caused an outbreak of hospital acquired respiratory tract infections [116].

The advantage of a literature survey is that published data is readily available and the meta-regression methods for analysing these types of data are established. In contrast to multi-country incidence studies, which tend to be a snap shot over typically less than six months, most of the studies here extended over more than twelve months. Here a random effects methods is used. By using this method, the precision associated with each individual study estimate is incorporated in the derivation of both the overall summary estimate and in the derivation of the meta-regression model. Moreover, in contrast to a fixed effects model, a random effect meta-regression model will generate more conservative summary estimates (i.e. wider 95 % confidence limits) as the method incorporates both within and between study variability. In this way, comparisons to address questions of study specific [115] and contextual [117] influences that would not be apparent within a single center study are enabled. As an example, the use of meta-regression can be used to benchmark control group pneumonia [112] and bacteremia [118] incidences in published prevention studies of VAP. The finding here of variability in incidence by region raises the possibility of contextual factors behind the variation.

There are several limitations to this literature based study. This is an analysis at the group level and is unable to take account of patient specific risk factors for *Acinetobacter* species associated VAP. For example, the usage of empiric antibiotic therapy in each study is an important unknown as use or non-use may account for vulnerability to *Acinetobacter* species associated VAP at the level of the individual patient [119, 120].

The grouping of countries into near neighbour groupings is somewhat arbitrary. Country and even regional groupings could be confounded by other variables such as infection control practices, prevalence of antibiotic use and standards of care for patients receiving mechanical ventilation that have not been able to be considered in the analysis here. Another limitation and difficult to exclude bias is the possible influence of publication bias.

The main limitation of a literature based survey is the lack of standardization across jurisdictions. It could be anticipated that there might be a range of clinical definitions used in the diagnosis of VAP at the level of the individual patient. That the mode of VAP diagnosis was not a significant factor in the regression model (Table 2) implies that this bias is likely to be minimal within a group level analysis as here. Likewise, the possibility of a linear time trend has been considered within the meta-regression model but this does not exclude the impact of trends more complex than linear.

An additional limitation is that for some reports, the VAP associated *Acinetobacter*, being the number of patients with VAP having an *Acinetobacter* species isolated from respiratory sampling, was not available. For these reports this number was derived as the number of patients with VAP multiplied by the proportion of VAP isolates that were *Acinetobacter* species. This is likely a reasonable approximation for a relatively rare outcome as found here. This approximation allows for VAP patients with multiple isolates.

A more difficult issue is that of laboratory documentation and reporting of Acinetobacter species type across jurisdictions. The striking observation among this survey was that all studies reported only one classification type of Acinetobacter. This was apparent in even the surveys with the most number of isolates [4, 27, 63]. The most common mode of reporting was as Acinetobacter species. Acinetobacter infections were less commonly reported as *Acinetobacter baumanii* from studies outside of Northern European and North American centers. It is possible that second line Acinetobacter species had been identified and listed within the category of ‘other’ gram negative infections. However, it remains a plausible explanation that the common practice in the literature reported here of the listing of a single Acinetobacter species generally reflects the mode of local reporting practices. In this regard and of pertinence to this survey of *Acinetobacter baumanii*, the reports of Acinetobacter species cannot be easily dismissed.

Of particular note, the rates of Acinetobacter VAP reported from studies reporting as Acinetobacter species versus studies reporting as *Acinetobacter baumanii* showed similar patterns of regional variation despite similar rates of overall VAP infection. Moreover, this regional variation in rates of Acinetobacter VAP were not explainable in a meta-regression model by trauma ICU, year of publication and Acinetobacter reporting practice as group level variables whereas a comparable meta-regression model of VAP showed no major regional variation in overall VAP rates (Additional file 1: Table S7).

The methods in use in clinical microbiology laboratories likely varied not only geographically but also likely temporally over the three decades encompassed in this survey [121–125]. Because of the limitations of the traditional phenotypic testing methods [121, 122] for identification, a broad category of *Acinetobacter calcoaceticus-A. baumanii* complex was suggested at one point [121]. These phenotypic methods are being superseded by newer and more specific molecular methods [123–125]. Moreover, these and even newer methods such as MALDI-TOF for microbial detection and identification will likely further refine the identification and reporting of Acinetobacter species clinical isolates going forward.

The lower prevalence of *Acinetobacter* in cooler seasons [106–108] is consistent with the finding here that the prevalence is lower in reports from countries further away from the equator. The biological mechanism for this difference remains speculative. However, *Acinetobacter* species are gram negative bacteria which have important additional international public health issue for two further reasons. The transportation of patients around the world create the potential for infection control failures [111]. Moreover, *Acinetobacter* species are commonly multi-drug resistant although rates of resistance vary from region to region [126].

## Conclusion

There is a greater than fivefold variation in *Acinetobacter* associated VAP among published reports from various geographic regions worldwide. This variation is not explainable by variations in rates of VAP overall, admissions for trauma, publication year or Acinetobacter reporting practices as group level variables.

## Additional file

Additional file 1: Tables of study data, a meta-regression of VAP incidence and listing of 100 references. (PDF 740 kb)

## Abbreviations

ICU: Intensive Care Unit
MV: Mechanical ventilation
VAP: Ventilator associated pneumonia
