# Supplementary material for: World-wide variation in incidence of Acinetobacter associated ventilator associated pneumonia: a meta-regression
Source: BMC Infect Dis. 2016 Oct 18;16:577. doi: 10.1186/s12879-016-1921-4 (PMC5070388; doi:10.1186/s12879-016-1921-4)
Supplement: Additional file 1: — Tables of study data, a meta-regression of VAP incidence and listing of 100 references. (PDF 740 kb) [file 12879_2016_1921_MOESM1_ESM.pdf]

Additional file: Table of contents

Table S1: Listing of studies (Multinational)	2
Table S2: Listing of studies (Northern Europe)	3 - 4
Table S3: Listing of studies (Mediterranean)	5
Table S4: Listing of studies (Asia & Middle East)	6
Table S5: Listing of studies (Americas)	7
Table S6: Listing of studies (Ungrouped)	8
Table S7: Log VAP incidence per thousand MV days; meta-regression model	9
References (s1-s100)	10 - 15

Abbreviations for additional file

CI; confidence interval

ICU, Intensive care unit;

MV; Mechanical ventilation

MVP; Percent of patients receiving mechanical ventilation

VAP; Ventilator associated pneumonia

Table S1: Listing of studies (Multinational)

Author	Ref	Notes*	mvp	Mean MV (days)	Total MV (days)	Patients (n)	VAP (n)	Acinetobacter (n - type†)
**Multinationa‡**								
Chevret et al 1993	S1	S	100	5	1275	255	55	5 Asp
Magret et al 2010	S2		100	5	10410	2082	337	42 Ab
Magret et al 2010	S2	T	100	5	1770	354	128	30 Ab
Rosenthal et al 2006	S3		100		9442	8867	284	43 Asp
Rosenthal et al 2006	S3		100		6376	1029	135	38 Asp
Rosenthal et al 2006	S3		100		8593	2172	86	3 Asp
Rosenthal et al 2006	S3		100		3401	3413	67	0 Asp
Rosenthal et al 2006	S3		100		3754	1514	73	2 Asp
Rosenthal et al 2006	S3		100		835	410	44	20 Asp
Rosenthal et al 2006	S3		100		3364	1359	98	5 Asp
Rosenthal et al 2006	S3		100		17222	2305	490	137 Asp
Rosenthal et al 2012	S4		100		10292	3889	226	43 Asp
Rosenthal et al 2012	S4	I	100		127347	51618	2191	426 Asp

Footnotes

* Group composition: T, Trauma; I Infection control intervention; S, Survey of less than 12 months

† Acinetobacter type; Ab, *Acinetobacter baumanii*; Asp, Acinetobacter species

‡Chevret et al 1993 (s1) is derived from a survey of ICU's in the following countries; France, Belgium, Italy, Germany, Spain, Portugal, Switzerland, Luxemburg, Denmark, Yugoslavia, United Kingdom, Netherlands, Austria, Greece, Hungary, Israel, Arabia, and Australia.

‡Magret et al 2010 (s2) is derived from a survey of ICU's in the following countries; France, Belgium, Italy, Germany, Greece, Ireland, Portugal, Spain, and Turkey.

‡Rosenthal et al 2006 (s3) is derived from a survey of ICU's in the following countries; Argentina, Brazil, Columbia, India, Mexico, Morocco, Peru, and Turkey.

‡Rosenthal et al 2012 (s4) is derived from a survey of ICU's in the following countries; Argentina, Brazil, China, Columbia, Costa Rica, Cuba, India, Lebanon, Macedonia, Mexico, Morocco, Panama, Peru, and Turkey.

Table S2: Listing of studies – **Northern Europe**

Author	Ref	Notes*	mvp	Mean MV (days)	Total MV (days)	Patients (n)	VAP (n)	Acinetobacter (n - type†)
**France**								
Bercault et al 2005	S5		100	9	1062	118	31	2 Ab
Bercault et al 2005	S5	I	100	11	1298	118	12	3 Ab
Bornstain et al 2004	S6	B	100	8.5	6350	747	80	1 Ab
Bregeon et al 1997	S7	B	100	10.3	6798	660	223	10 Asp
Chastre et al 1998	S8	B	100	15	2805	187	53	7 Ab
Fagon et al 1989	S9	B	100	13	7371	567	49	8 Asp
Garrouste-Orgas 1997	S10	B	100	11	946	86	31	11 Ab
Georges et al 2000	S11	B	100	18	2430	135	35	8 Ab
Gruson et al 2000	S12	B, 95/96	100	11.6	11646	1004	231	20 Ab
Gruson et al 2000	S12	B, 97/98	100	11.9	12245	1029	161	7 Ab
Gruson et al 2003	S13	B, 99/01	100	11.6	9547	823	134	15 Ab
Guerin et al 1997	S14	B	100	8.9	2314	260	27	1 Ab
Jailette et al 2011	S15		100	15	6585	439	137	22 Ab
Luyt et al 2005	S16		100	35	10150	290	69	3 Ab
Mahul et al 1992	S17	B	100	16.6	2407	145	30	3 Asp
Markowicz et al 2000	S18	B	100	11.7	8705	744	162	13 Ab
Moine et al 2002	S19	B	80	9.7	7411	764	89	1 Ab
Nseir et al 2005	S20		100	24	29784	1241	77	20 Ab
Papazian et al 1996	S21	B	100	16	9376	586	97	5 Asp
Stéphan et al 2006	S22	T	100	5	875	175	78	8 Ab
Timsit et al 1996	S23	B	100	12	4644	387	56	8 Ab
Trouillet et al 1998	S24		100	17	8466	498	135	22 Ab

* Group composition: I Infection control intervention; B, bronchoscopic techniques for isolate sampling; C, cardiac; T, Trauma

† Acinetobacter type; Ab, *Acinetobacter baumanii*; Asp, Acinetobacter species;

Table S2: Listing of studies - **Northern Europe** (continued)

Author	Ref	Notes*	mvp	Mean MV (days)	Total MV (days)	Patients (n)	VAP (n)	Acinetobacter (n - type†)
**Germany, Netherlands, Belgium, Denmark**								
Daschner et al 1988	S25		100	6	852	142	68	23 Asp
Daschner et al 1988	S26		100	6	696	116	36	1 Asp
Kohlenberg et al 2000	S27		100		1068472	779500	5811	152 Asp
Myny et al 2005	S28		100	6	2310	385	89	13 Ab
Nielsen et al 1992	S29		100	3.8	919.6	242	23	0
Verhamme et al 2007	S30	B	84	7.7	22230	4000	298	3 Asp
Woske et al 2001	S31	B	100	15	1545	103	49	1 Asp
**UK, Scandinavia, Eastern Europe, Switzerland**								
A'court et al 1993	S32	T	100	9	1350	150	33	0
Duszyńska et al 2015	S33		100		8425	1097	93	48 Ab
Hugonnet et al 2007	S34	B	100	4.8	4493	936	209	8 Asp
Hyllienmark et al 2007	S35	B	100	5	1105	221	33	1 Asp
Magnason et al 2008	S36		100		933	280	21	0
Reusser et al 1989	S37		100	7	280	40	15	1 Asp

* Group composition: B, bronchoscopic techniques for isolate sampling; C, cardiac; T, Trauma

† Acinetobacter type; Ab, *Acinetobacter baumanii*; Asp, Acinetobacter species

Table S3: Listing of studies - **Mediterranean**

Author	Ref	Notes*	mvp	Mean MV (days)	Total MV (days)	Patients (n)	VAP (n)	Acinetobacter (n - type†)
**Spain**								
Alvarez-Lerma et al 1996	S38	B	93	9.2	59745	6494	519	56 Asp
Baraibar et al 1997	S39	B	100	8	5656	707	148	12 Ab
Cavalcanti et al 2006	S40	B, T	100	5	950	190	62	1 Ab
Cenderero et al 1999	S41	B	100	6.5	799	123	19	2 Ab
de Latore et al 1995	S42	B	100	10.2	816	80	12	0
Ewig et al 1999	S43	B, T	100	6	288	48	10	0
Jimenez et al 1989	S44	S	100	5.6	431	77	18	6 Asp
Rello et al 1991	S45	B	100	7.9	2086	264	58	2 Ac
Rello et al 2003	S46	B	100	7	693	99	18	2 Ab
Rincón-Ferrari et al 2004	S47	B, T	100	10	3100	310	72	27 Asp
Ruiz-Santana et al 1987	S48	B	100	7	7035	1005	180	0
Tamayo et al 2012	S49	C	100		5799	1610	124	35 Asp
Tejada-Artigas et al 2001	S50	B, T	100	8	824	103	23	10 Ab
Torres et al 1990	S51		100	4	1288	322	78	9 Ac
Violan et al 1998	S52	B	100	13	4082	314	82	1 Ab
**Italy/Greece/Tunisia**								
Antonelli et al 1994	S53	B, T	67	11.9	1476	124	41	6 Aa
Apostolopoulou 2003	S54	S	100	12.6	2205	175	56	
Kallel et al 2005	S55	T	100	13.7	3302	241	77	31 Ab
Piazza et al 2006	S56		100	17.2	2466	143	29	17 Ab
Sofianou et al 2000	S57		100		1584	198	67	35 Asp

Footnotes

* Group composition: B, bronchoscopic techniques for isolate sampling; C, cardiac; T, Trauma; S, Survey of less than 12 months

† Acinetobacter type; Ab, *Acinetobacter baumanii*; Asp, Acinetobacter species; Ac *Acinetobacter calcoaceticus;* Aa *Acinetobacter anitratus*

Table S4: Listing of studies – Asia & Middle East

Author	Ref	Notes*	mvp	Mean MV (days)	Total MV (days)	Patients (n)	VAP (n)	Acinetobacter (n - type†)
**Asia‡**								
Mallick et al 2015	S58		100		1707	301	61	26 Ab
Noor et al 2005	S59		100	6	1500	250	70	15 Asp
Pawar et al 2003	S60	C, S	100		919	952	25	2 Asp
Rozaidi et al 2001	S61	B	65		3944	988	53	12 Asp
Tan et al 2016	S62		100		5562	618	286	114 Ab
Tao et al 2011	S63		100		540536	391527	11224	1562 Ab
Tao et al 2012	S64		100		29041	16426	365	60 Ab
**Middle East‡**								
Akça et al 2000	S65		100	7.1	1846	260	81	9 Asp
Alp et al 2004	S66	T	100	3.9	3128	724	123	42 Ab
Ertugrul et al 2006	S67	T	100	9.7	970	100	28	6 Asp
Gursel et al 2010	S68		100	10	920	92	59	23 Ab
Kanafani et al 2003	S69	S	100	17	1190	70	33	11 Aa
Landrum et al 2008	S70	T	100		231		14	7 Asp
Landrum et al 2008	S70	T, I	100		526		11	7 Asp
Leblebicioglu et al 2007	S71		100		23520	3296	623	182 Asp
Leblebicioglu et al 2013	S72		100		28181	3864	474	130 Asp
Leblebicioglu et al 2013	S72	I	100		2376	448	74	12 Asp
Memish et al 2000	S73		100	8	3035	202	41	14 Asp

Footnotes

* Group composition: B, bronchoscopic techniques for isolate sampling; C, cardiac; T, Trauma; I Infection control intervention; S, Survey of less than 12 months

† Acinetobacter type; Ab, *Acinetobacter baumanii*; Asp, Acinetobacter species; Ac *Acinetobacter calcoaceticus;* Aa *Acinetobacter anitratus*

‡Asia includes China, India, Bangladesh and Pakistan.

‡Middle East includes Turkey, Lebanon, Iraq, and Saudi Arabia.

Table S5: Listing of studies - Americas

Author	Ref	Notes*	mvp	Mean MV (days)	Total MV (days)	Patients (n)	VAP (n)	Acinetobacter (n - type†)
**Central & South America‡**								
Berg et al 1995	S74	S	54		888	253	62	11 Asp
Guanche-Garcell 2013	S75		100		114	67	6	1 Asp
Guanche-Garcell 2013	S75	I	100		2350	1008	36	1 Asp
Guimaraes et al 2006	S76		100	7	1946	278	106	8 Asp
Jaimes et al 2007	S77		100	9.6	2592	270	60	2 Ab
Luna et al 2003	S78	B	100	7.6	3245	427	63	25 Ab
Moreno et al 2006	S79		100		8593	2172	86	2 Asp
Resende et al 2013	S80		100	24	3024	126	33	11 Asp
Rodrigues et al 2009	S81		100	10	2330	233	64	14 Ab
**USA & Canada**								
Baker et al 1996	S82	B, T	100		5140	514	30	6 Asp
Beck-Sague et al 1996	S83	T, S	100	5	725	145	15	0
Bochicchio et al 2004	S84	T	100	10.5	7119	678	125	10 Asp
Cook et al 2010	S85	B	100	5.5	11440	2080	70	1 Ab
Cook et al 2010	S85	B, T	100	7.3	3730	511	91	8 Ab
Craven et al 1988	S86	M	100	4	1108	277	47	0
Craven et al 1988	S86	S	100	3.6	1876	521	49	0
Ensminger et al 2006	S87	C	100		2135	92	17	1 Asp
George et al 1998	S88	B	100	6	1338	223	28	1 Asp
Heyland et al 1999	S89	B	100	16	16224	1014	177	6 Asp
Ibrahim et al 2000	S90		100	4.7	8845	1882	397	16 Asp
Kollef et al 1993	S91	S	100	7.4	2050	277	43	1 Ac
Kollef et al 1995	S92	S	100			314	87	4 Asp
Kollef et al 1997	S93	C	100	2.5	883	353	42	2 Ab
Kollef et al 1997	S93	C, I	100	2.4	785	327	23	1 Ab
Kunac et al 2014	S94	B, T	100	5	3580	716	206	13 Ab
Rodriguez et al 1991	S95	T	100	11	3234	294	130	11 Asp
Salata et al 1987	S96	B	100	13.5	688	51	21	0

Footnotes

* Group composition: B, bronchoscopic techniques for isolate sampling; C, cardiac; T, Trauma; I Infection control intervention; S, Survey of less than 12 months

† Acinetobacter type; Ab, *Acinetobacter baumanii*; Asp, Acinetobacter species; Ac *Acinetobacter calcoaceticus;*

‡Central & South America includes Argentina, Brazil, Chile, Colombia, Cuba, Peru, and Mexico.

Table S6: Listing of studies – Ungrouped

Author	Ref	Notes*	mvp	Mean MV (days)	Total MV (days)	Patients (n)	VAP (n)	Acinetobacter (n - type†)
**Australia/ South Africa**								
Behari et al 2015	S97	S	100		808	32	8	3 Ab
Potgieter et al 1987	S98	S	78	9.2	2300	250	51	32 Asp
Boots et al 2008	S99	B	100	10.6	4367	412	58	14 Ac
Cade et al 1993	S100		98	4	392	98	35	0

Footnotes

* Group composition: B, bronchoscopic techniques for isolate sampling; S, Survey of less than 12 months

† Acinetobacter type; Ab, *Acinetobacter baumanii*; Asp, Acinetobacter species; Ac *Acinetobacter calcoaceticus;*

Table S7: Log VAP incidence per thousand MV days; meta-regression model [a]

	Studies reporting as either Acinetobacter species or *Acinetobacter baumanii*			Only studies reporting as *Acinetobacter baumanii*		
Factor	Coefficient [b]	95% CI	p	Coefficient [b]	95% CI	p
Northern European studies (reference group)	3·46	2·92 - +4·0		3·08	2·31 - +3·85	
Geographic region						
• Mediterranean	+0·37	-0·07 - +0·80	0·095	+0·41	-0·13 - +0·95	0·13
• Asia	+0·16	-0·54 - +0·87	0·65	+0·17	-0·83 - +1·16	0·74
• Middle East	+0·43	-0·13 - +0·98	0·13	+0·96	+0·01 - +1·9	0·05
• Central & South America	+0·54	-0·13 - +1·21	0·11	+0·39	-0·54 - +1·32	0·40
• USA & Canada	-0·08	-0·52 - +0·37	0·74	+0·30	-0·29 - +0·90	0·31
• Ungrouped	+0.49	-0·47 - +1.45	0.31	+1.60	+0·26 - +2.9	0.02
• Multinational	+0.40	-0·10 - +0.90	0.12	+1.02	+0·04 - +2.0	0.04
Trauma [c]	+0·39	+0·01 - +0.78	0·05	+0·64	+0·12 - +1.16	0·02
Year of publication [d]	-0·03	-0·04 - +0·01	0·06	-0·01	-0·05 - +0·02	0·34
Mode of diagnosis [e]	-0·22	-0·54 - +0·09	0·16	-0·10	-0·52 - +0·32	0·63
Intervention period [f]	-0.18	-0.82 - +0.46	0.58	+0.12	-0.89 - +1.1	0.81
Acinetobacter baumanii [g]	+0·03	-0·28 - +0·33	0·87			

Footnotes

a. This table displays the results of a meta-regression analysis for log VAP incidence per thousand MV days.
b. Interpretation. The reference group is the Northern European studies and this coefficient equals the difference in log from 0 (a log equal to 0 equates to a rate of 1. The other coefficients represent the difference in log for groups positive for that factor versus the reference group.
c. The co-efficient for trauma represents the increment in log for an ICU having a majority of admissions for trauma
d. Year of publication co-efficient represents the linear increment in log for each year after 1980
e. For sampling using bronchoscopic versus tracheal sampling
f. Studies undertaken during an infection control intervention
g. Studies reporting Acinetobacter infections as *Acinetobacter baumanii* versus reporting as Acinetobacter species or otherwise.

References

S1. Chevret S, Hemmer M, Carlet J, et al. Incidence and risk factors of pneumonia acquired in intensive care units. Results from a multicenter prospective study on 996 patients. European Cooperative Group on Nosocomial Pneumonia. Intensive Care Med. 1993;19:256-264.

S2. Magret M, Amaya-Villar R, Garnacho J, Lisboa T, Diaz E, DeWaele J, Deja M, Manno E, Rello J, EU-VAP/CAP Study Group. Ventilator-associated pneumonia in trauma patients is associated with lower mortality: results from EU-VAP study. J Trauma Acute Care Surg. 2010;69(4):849-54.

S3. Rosenthal VD, Maki DG, Salomao R, Moreno CA, Mehta Y, Higuera F, Cuellar LE, Arikan OA, Abouqal R, Leblebicioglu H. Device-associated nosocomial infections in 55 intensive care units of 8 developing countries. Ann Intern Med. 2006;145(8):582-91.

S4. Rosenthal VD, Rodrigues C, Madani N, Mitrev Z, Ye G, Salomao R, Ulger F, Guanche-Garcell H, Kanj SS, Cuéllar LE, Higuera F. Effectiveness of a multidimensional approach for prevention of ventilator-associated pneumonia in adult intensive care units from 14 developing countries of four continents: Findings of the International Nosocomial Infection Control Consortium. Crit Care Med. 2012;40(12):3121-8.

S5. Bercault N, Wolf M, Runge I, et al. Intrahospital transport of critically ill ventilated patients: a risk factor for ventilator-associated pneumonia--a matched cohort study. Crit Care Med. 2005;33:2471-8.

S6. Bornstain C, Azoulay E, De Lassence A, Cohen Y, Costa MA, Mourvillier B, Descorps-Declere A, Garrouste-Orgeas M, Thuong M, Schlemmer B, Timsit JF. Sedation, sucralfate, and antibiotic use are potential means for protection against early-onset ventilator-associated pneumonia. Clin Infect Dis. 2004;38(10):1401-8.

S7. Bregeon F, Papazian L, Visconti A, Gregoire R, Thirion X, Gouin F. Relationship of microbiologic diagnostic criteria to morbidity and mortality in patients with ventilator-associated pneumonia. JAMA. 1997,277: 655-662.;

S8. Chastre J, Trouillet JL, Vuagnat A, et al. Nosocomial pneumonia in patients with acute respiratory distress syndrome. Am J Respir Crit Care Med 1998;157:1165-72.

S9. Fagon JY, Chastre J, Domart Y, Trouillet JL, Pierre J, Darne C, Gibert C. Nosocomial pneumonia in patients receiving continuous mechanical ventilation. Prospective analysis of 52 episodes with use of a protected specimen brush and quantitative culture techniques. Am Rev Respir Dis. 1989; 139:877-884.

S10. Garrouste-Orgeas M, Chevret S, Arlet G, et al. Oropharyngeal or gastric colonization and nosocomial pneumonia in adult intensive care unit patients. A prospective study based on genomic DNA analysis. Am J Respir Crit Care Med. 1997;156:1647-56.

S11. Georges H, Leroy O, Guery B, Alfandari S, Beaucaire G. Predisposing factors for nosocomial pneumonia in patients receiving mechanical ventilation and requiring tracheotomy. Chest. 2000;118:767–774.

S12. Gruson D, Hilbert G, Vargas F, Valentino R, Bebear C, Allery A, Bebear C, Gbikpi-benissan GE, Cardinaud JP. Rotation and restricted use of antibiotics in a medical intensive care unit: impact on the incidence of ventilator-associated pneumonia caused by antibiotic-resistant gram-negative bacteria. Am J Respir Crit Care Med. 2000;162(3):837-43.

S13. Gruson D, Hilbert G, Vargas F, et al. Strategy of antibiotic rotation: long-term effect on incidence and susceptibilities of Gram-negative bacilli responsible for ventilator-associated pneumonia. Crit Care Med. 2003;31:1908-1914.

S14. Guerin C, Girard R, Chemorin C, De Varax R, Fournier G. Facial mask noninvasive mechanical ventilation reduces the incidence of nosocomial pneumonia. Intensive care Med. 1997;23(10):1024-32.

S15. Jaillette E, Nseir S. Relationship between inhaled β2-agonists and ventilator-associated pneumonia: A cohort study. Critical Care Med. 2011;39(4):725-30.

S16. Luyt CE, Guerin V, Combes A, et al. Procalcitonin kinetics as a prognostic marker of ventilator-associated pneumonia. Am J Respir Crit Care Med. 2005;171:48-53.

S17. Mahul P, Auboyer C, Jospe R, Ros A, Guerin C, el Khouri Z, Galliez M, Dumont A, Gaudin O. Prevention of nosocomial pneumonia in intubated patients respective role of mechanical subglottic secretions drainage and stress ulcer prophylaxis. Intensive Care Med. 1992;18:20-25.

S18. Markowicz P, Wolff M, Djedaini K, Cohen Y, Chastre J, Delclaux C. Multicenter prospective study of ventilator-associated pneumonia during acute respiratory distress syndrome. Incidence, prognosis, and risk factors. ARDS Study Group. Am J Respir Crit Care Med. 2000;161:1942-8.

S19. Moine P, Timsit JF, De Lassence A, et al. Mortality associated with late-onset pneumonia in the intensive care unit: results of a multi-center cohort study. Intensive Care Med. 2002;28:154-63.

S20. Nseir S, Di Pompeo C, Soubrier S, Cavestri B, Jozefowicz E, Saulnier F, Durocher A. Impact of ventilator-associated pneumonia on outcome in patients with COPD. Chest. 2005;128(3):1650-6.

S21. Papazian L, Bregeon F, Thirion X, et al. Effect of ventilator-associated pneumonia on mortality and morbidity. Am J Respir Crit Care Med. 1996;154:91-7.

S22. Stéphan F, Mabrouk N, Decailliot F, Delclaux C, Legrand P. Ventilator-associated pneumonia leading to acute lung injury after trauma: importance of Haemophilus influenzae. Anesthesiol. 2006;104:235-41.

S23. Timsit JF, Chevret S, Valcke J, et al. Mortality of nosocomial pneumonia in ventilated patients: influence of diagnostic tools. Am J Respir Crit Care Med. 1996;154:116-23.

S24. Trouillet JL, Chastre J, Vuagnat A, Joly-Guillou ML, Combaux D, Dombret MC, Gibert C. Ventilator-associated pneumonia caused by potentially drug-resistant bacteria. Am J Respir Crit Care Med. 1998;157(2):531-9.

S25. Daschner F, Kappstein I, Schuster F, et al. Influence of disposable ('Conchapak') and reusable humidifying systems on the incidence of ventilation pneumonia. J Hosp Infect. 1988;11:161-168

S26. Daschner F, Kappstein I, Engels I, Reuschenbach K, Pfisterer J, Krieg N, Vogel W. Stress Ulcer Prophylaxis and Ventilation Pneumonia Prevention by Antibacterial Cytoprotective Agents? Infection Control. 1988;9(02):59-65.

S27. Kohlenberg A, Schwab F, Behnke M, Geffers C, Gastmeier P. Pneumonia associated with invasive and noninvasive ventilation: an analysis of the German nosocomial infection surveillance system database. Intensive Care Med. 2010;36(6):971-8.

S28. Myny D, Depuydt P, Colardyn F, Blot S. Ventilator-associated pneumonia in a tertiary care ICU analysis of risk factors for acquisition and mortality. Acta Clin Belg. 2005;60:114-121.

S29. Nielsen SL, Roder B, Magnussen P, et al. Nosocomial pneumonia in an intensive care unit in a Danish university hospital: incidence, mortality and etiology. Scand J Infect Dis. 1992;24:65-70.

S30. Verhamme KM, De Coster W, De Roo L, De Beenhouwer H, Nollet G, Verbeke J, Demeyer I, Jordens P. Pathogens in early-onset and late-onset intensive care unit–acquired pneumonia. Infection Control & Hospital Epidemiol. 2007;28(04):389-97.

S31. Woske HJ, Röding T, Schulz I, Lode H. Ventilator-associated pneumonia in a surgical intensive care unit Epidemiology, etiology and comparison of three bronchoscopic methods for microbiological specimen sampling. Crit Care. 2001;5:167–173.

S32. A'Court CH, Garrard CS, Crook D, et al. Microbiological lung surveillance in mechanically ventilated patients, using non-directed bronchial lavage and quantitative culture. Q J Med. 1993;86:635-48.

S33. Duszyńska W, Rosenthal VD, Dragan B, Węgrzyn P, Mazur A, Wojtyra P, Tomala A, Kübler A. Ventilator-associated pneumonia monitoring according to the INICC project at one centre. Anaesthesiology Intensive Ther. 2015;47(1):34-9.

S34. Hugonnet S, Uçkay I, Pittet D. Staffing level: a determinant of late-onset ventilator-associated pneumonia. Crit Care. 2007;11(4), R80.

S35. Hyllienmark P, Gardlund B, Persson JO, Ekdahl K. Nosocomial pneumonia in the ICU: a prospective cohort study. Scand J Infect Dis. 2007;39:676-82.

S36. Magnason S, Kristinsson KG, Stefansson T, Erlendsdottir H, Jonsdottir K, Kristjansson M, Gudmundsson S. Risk factors and outcome in ICU-acquired infections. Acta Anaesthesiologica Scand. 2008;52:1238-1245.

S37. Reusser P, Zimmerli W, Scheidegger D, Marbet GA, Buser M, Gyr K. Role of gastric colonization in nosocomial infections and endotoxemia: a prospective study in neurosurgical patients on mechanical ventilation. J Infect Dis. 1989;160:414-421.

S38. Alvarez-Lerma F, ICU-acquired Pneumonia Study Group. Modification of empiric antibiotic treatment in patients with pneumonia acquired in the intensive care unit. Intensive Care Med. 1996;22(5):387-94.

S39. Baraibar J, Correa H, Mariscal D, Gallego M, Valles J, Rello J. Risk factors for infection by Acinetobacter baumannii in intubated patients with nosocomial pneumonia. Chest. 1997;112(4):1050-4.

S40. Cavalcanti M, Ferrer M, Ferrer R, et al. Risk and prognostic factors of ventilator-associated pneumonia in trauma patients. Crit Care Med. 2006;34:1067-1072.

S41. Cardenosa Cendrero JA, Sole-Violan J, Bordes Benitez A, et al. Role of different routes of tracheal colonization in the development of pneumonia in patients receiving mechanical ventilation. Chest. 1999;116:462-470.

S42. de Latorre FJ, Pont T, Ferrer A, et al. Pattern of tracheal colonization during echanical ventilation. Am J Respir Crit Care Med. 1995;152:1028-1033.

S43. Ewig S, Torres A, El-Ebiary M, et al. Bacterial colonization patterns in mechanically ventilated patients with traumatic and medical head injury. Incidence, risk factors, and association with ventilator-associated pneumonia. Am J Respir Crit Care Med. 1999;159:188-198.

S44. Jimenez P, Torres A, Rodriguez-Roisin R, et al. Incidence and etiology of pneumonia acquired during mechanical ventilation. Crit Care Med. 1989;17:882-5.

S45. Rello J, Quintana E, Ausina V, et al. Incidence, etiology, and outcome of nosocomial pneumonia in mechanically ventilated patients. Chest. 1991;100:439-44.

S46. Rello J, Lorente C, Diaz E, et al. Incidence, etiology, and outcome of nosocomial pneumonia in ICU patients requiring percutaneous tracheotomy for mechanical ventilation. Chest. 2003;124:2239-2243.

S47. Rincón-Ferrari MD, Flores-Cordero JM, Leal-Noval SR, Murillo-Cabezas F, Cayuelas A, Muñoz-Sánchez MA, Sánchez-Olmedo JI. Impact of ventilator-associated pneumonia in patients with severe head injury. J Trauma Acute Care Surg. 2004;57(6):1234-40.

S48. Ruiz-Santana S, Garcia Jimenez A, Esteban A, et al. ICU pneumonias: a multi-institutional study. Crit Care Med. 1987;15:930-932.

S49. Tamayo E, Álvarez FJ, Martínez-Rafael B, Bustamante J, Bermejo-Martin JF, Fierro I, Eiros JM, Castrodeza J, Heredia M, Gómez-Herreras JI, Valladolid Sepsis Study Group. Ventilator-associated pneumonia is an important risk factor for mortality after major cardiac surgery. J Crit Care. 2012;27(1):18-25.

S50. Tejada Artigas A, Bello Dronda S, Chacon Valles E, et al. Risk factors for nosocomial pneumonia in critically ill trauma patients. Crit Care Med. 2001;29:304-9.

S51. Torres A, Aznar R, Gatell JM, et al. Incidence, risk, and prognosis factors of nosocomial pneumonia in mechanically ventilated patients. Am Rev Respir Dis. 1990;142:523-8.

S52. Violan JS, Sanchez-Ramirez C, Mujica AP, Cendrero JC, Fernandez JA, de Castro FR. Impact of nosocomial pneumonia on the outcome of mechanically-ventilated patients. Crit Care (Lond). 1998;2:19-23.

S53. Antonelli M, Moro ML, Capelli O, et al. Risk factors for early onset pneumonia in trauma patients. Chest. 1994;105:224-228.

S54. Apostolopoulou E, Bakakos P, Katostaras T, et al. Incidence and risk factors for ventilator-associated pneumonia in 4 multidisciplinary intensive care units in Athens, Greece. Respir Care. 2003;48:681-8.

S55. Kallel H, Chelly H, Bahloul M, Ksibi H, Dammak H, Chaari A, Hamida CB, Rekik N, Bouaziz M. The effect of ventilator-associated pneumonia on the prognosis of head trauma patients. J Trauma Acute Care Surg. 2005;59(3):705-10.

S56. Piazza O, Iasiello A, PapaIanni C, De Robertis E, Servillo G, Rossano F, Tufano R. Incidence of antimicrobial-resistant ventilator associated pneumonia: an eighteen-month survey. *Panminerva medica.* 2005 47(4):265-7.

S57. Sofianou DC, Constandinidis TC, Yannacou M, et al. Analysis of risk factors for ventilator-associated pneumonia in a multidisciplinary intensive care unit. Eur J Clin Microbiol Infect Dis. 2000;19:460-3.

S58. Mallick UK, Faruq MO, Ahsan AA, Fatema K, Ahmed F, Asaduzzaman M, Islam M, Sultana A. Spectrum of Early Onset and Late Onset Ventilator Associated Pneumonia (VAP) in a Tertiary Care Hospital of Bangladesh: A Prospective Cohort Study. Bangladesh Crit Care J. 2015;3(1):9-13.

S59. Noor A, Hussain SF. Risk factors associated with development of ventilator associated pneumonia. J Coll Physicians Surg Pak. 2005;15:92–95.

S60. Pawar M, Mehta Y, Khurana P, Chaudhary A, Kulkarni V, Trehan N. Ventilator-associated pneumonia: incidence, risk factors, outcome, and microbiology. J Cardiothoracic Vascular Anesthesia. 2003;17(1):22-8.

S61. Rozaidi SW, Sukro J, Dan A. The incidence of nosocomial infection in the Intensive Care Unit, Hospital Universiti Kebangsaan Malaysia: ICU-acquired nosocomial infection surveillance program 1998-1999. Med J Malaysia. 2001;56(2):207-22.

S62. Tan X, Zhu S, Yan D, Chen W, Chen R, Zou J, Yan J, Zhang X, Farmakiotis D, Mylonakis E. Candida spp. airway colonization: A potential risk factor for Acinetobacter baumannii ventilator-associated pneumonia. Med Mycol. 2016:myw009.

S63. Tao L, Hu B, Rosenthal VD, Gao X, He L. Device-associated infection rates in 398 intensive care units in Shanghai, China: International Nosocomial Infection Control Consortium (INICC) findings. Int J Infect Dis. 2011;15(11):e774-80.

S64. Tao L, Hu B, Rosenthal VD, Zhang Y, Gao X, He L. Impact of a multidimensional approach on ventilator-associated pneumonia rates in a hospital of Shanghai: findings of the International Nosocomial Infection Control Consortium. J Crit Care. 2012;27(5):440-6.

S65. Akça O, Koltka K, Uzel S, et al. Risk factors for early-onset, ventilator-associated pneumonia in critical care patients: selected multiresistant versus non-resistant bacteria. Anesthesiol. 2000;93: 638-45.

S66. Alp E, Güven M, Yıldız O, Aygen B, Voss A, Doganay M. Incidence, risk factors and mortality of nosocomial pneumonia in intensive care units: a prospective study. Ann Clin Microbiol Antimicrob. 2004;3(1):1.

S67. Ertugrul BM, Yildirim A, Ay P, Oncu S, Cagatay A, Cakar N, Ertekin C, Ozsut H, Eraksoy H, Calangu S. Ventilator-associated pneumonia in surgical emergency intensive care unit. Saudi Med J. 2006;27(1):52-7.

S68. Gursel G, Aydogdu M, Nadir Ozis T, Tasyurek S. Comparison of the value of initial and serial endotracheal aspirate surveillance cultures in predicting the causative pathogen of ventilator-associated pneumonia. Scand J Infect Dis. 2010, 42:341-346.

S69. Kanafani ZA, Kara L, Hayek S, et al. Ventilator-associated pneumonia at a tertiary-care center in a developing country: incidence, microbiology, and susceptibility patterns of isolated microorganisms. Infect Control Hosp Epidemiol. 2003;24:864-9.

S70. Landrum ML, Murray CK. Ventilator associated pneumonia in a military deployed setting: the impact of an aggressive infection control program. J Trauma Acute Care Surg. 200;64(2):S123-8.

S71. Leblebicioglu H, Rosenthal VD, Arıkan ÖA, Özgültekin A, Yalcin AN, Koksal I, Usluer G, Sardan YC, Ulusoy S. Device-associated hospital-acquired infection rates in Turkish intensive care units. Findings of the International Nosocomial Infection Control Consortium (INICC). J Hosp Infect. 2007;65(3):251-7.

S72. Leblebicioglu H, Yalcin AN, Rosenthal VD, Koksal I, Sirmatel F, Unal S, Turgut H, Ozdemir D, Ersoz G, Uzun C, Ulusoy S. Effectiveness of a multidimensional approach for prevention of ventilator-associated pneumonia in 11 adult intensive care units from 10 cities of Turkey: findings of the International Nosocomial Infection Control Consortium (INICC). Infect. 2013;41(2):447-56.

S73. Memish ZA, Cunningham G, Oni GA, et al. The incidence and risk factors of ventilator-associated pneumonia in a Riyadh hospital. Infect Control Hosp Epidemiol. 2000;21:271-3.

S74. Berg DE, Hershow RC, Ramirez CA, Weinstein RA. Control of nosocomial infections in an intensive care unit in Guatemala City. Clin Infect Dis. 1995;21:588-593.

S75. Guanche-Garcell H, Morales-Perez C, Rosenthal VD. Effectiveness of a multidimensional approach for the prevention of ventilator-associated pneumonia in an adult intensive care unit in Cuba: findings of the International Nosocomial Infection Control Consortium (INICC). J Infect Public Health. 2013;6:98-107.

S76. Guimaraes MM, Rocco JR. Prevalence of ventilator-associated pneumonia in a university hospital and prognosis for the patients affected. J Bras Pneumol. 2006;32:339–346.

S77. Jaimes F, De La Rosa G, Gómez E, Múnera P, Ramírez J, Castrillón S. Incidence and risk factors for ventilator-associated pneumonia in a developing country Where is the difference? Respir Med. 2007;101:762–767.

S78. Luna CM, Blanzaco D, Niederman MS, et al. Resolution of ventilator-associated pneumonia: prospective evaluation of the clinical pulmonary infection score as an early clinical predictor of outcome. Crit Care Med. 2003;31:676-682.

S79. Moreno CA, Rosenthal VD, Olarte N, Gomez WV, Sussmann O, Agudelo JG, Rojas C, Osorio L, Linares C, Valderrama A, Mercado PG. Device-associated infection rate and mortality in intensive care units of 9 Colombian hospitals: findings of the International Nosocomial Infection Control Consortium. Infect Control. 2006;27(04):349-56.

S80. Resende MM, Monteiro SG, Callegari B, Figueiredo PM, Monteiro CR, Monteiro-Neto V. Epidemiology and outcomes of ventilator-associated pneumonia in northern Brazil: an analytical descriptive prospective cohort study. BMC Infect Dis. 2013;13(1):119.

S81. Rodrigues PM, Neto C, Santos LR, Knibel MF. Ventilator-associated pneumonia: epidemiology and impact on the clinical evolution of ICU patients. Jornal brasileiro de pneumologia. 2009 Nov;35(11):1084-91.

S82. Baker AM, Meredith JW, Haponik EF. Pneumonia in intubated trauma patients. Microbiology and outcomes. Am J Respir Crit Care Med. 1996;153:343-9.

S83. Beck-Sague CM, Sinkowitz RL, Chinn RY, et al. Risk factors for ventilator-associated pneumonia in surgical intensive-care-unit patients. Infect Control Hosp Epidemiol. 1996;17:374-6.

S84. Bochicchio GV, Joshi M, Bochicchio K, et al. A time-dependent analysis of intensive care unit pneumonia in trauma patients. J Trauma. 2004;56:296-301.

S85. Cook A, Norwood S, Berne J. Ventilator-associated pneumonia is more common and of less consequence in trauma patients compared with other critically ill patients. J Trauma Acute Care Surg. 2010;69(5):1083-91.

S86. Craven DE, Kunches LM, Lichtenberg DA, et al. Nosocomial infection and fatality in medical and surgical intensive care unit patients. Arch Intern Med. 1988;148:1161-8.

S87. Ensminger SA, Wright RS, Baddour LM, Afess B. Suspected ventilator-associated pneumonia in cardiac patients admitted to the coronary care unit. Mayo Clin. Proc. 2006;81:32–35.

S88. George DL, Falk PS, Wunderink RG, Leeper Jr KV, Meduri GU, Steere EL, Glen Mayhall C. Epidemiology of ventilator-acquired pneumonia based on protected bronchoscopic sampling. Am J Respir Crit Care Med. 1998;158:1839-1847.

S89. Heyland DK, Cook DJ, Griffith L, Keenan SP, Brun-Buisson C. The attributable morbidity and mortality of ventilator-associated pneumonia in the critically ill patient. The Canadian Critical Trials Group. Am J Respir Crit Care Med. 1999;159:1249-1256.

S90. Ibrahim EH, Ward S, Sherman G, Kollef MH. A comparative analysis of patients with early-onset vs late-onset nosocomial pneumonia in the ICU setting. Chest. 2000;117:1434-1442.

S91. Kollef MH. Ventilator-associated pneumonia. A multivariate analysis. JAMA. 1993;270:1965-70.

S92. Kollef MH, Silver P, Murphy DM, et al. The effect of late-onset ventilator-associated pneumonia in determining patient mortality. Chest. 1995;108:1655-62.

S93. Kollef MH, Vlasnik J, Sharpless L, Pasque C, Murphy D, Fraser V. Scheduled change of antibiotic classes A strategy to decrease the incidence of ventilator-associated pneumonia. Am J Respir Crit Care Med.1997;156:1040–1048.

S94. Kunac A, Sifri ZC, Mohr AM, Horng H, Lavery RF, Livingston DH. Bacteremia and Ventilator-Associated Pneumonia: A Marker for Contemporaneous Extra-Pulmonic Infection. Surg Infect. 2014;15:77-83.

S95. Rodriguez JL, Gibbons KJ, Bitzer LG, et al. Pneumonia: incidence, risk factors, and outcome in injured patients. J Trauma. 1991;31:907-12.

S96. Salata RA, Lederman MM, Shlaes DM, Jacobs MR, Eckstein E, Tweardy D, Toossi Z, Chmielewski R, Marino J, King CH. Diagnosis of nosocomial pneumonia in intubated, intensive care unit patients. Am Rev Respir Dis. 1987;135:426-432.

S97. Behari AA, Kalafatis N. Incidence and outcome of ventilator-associated pneumonia in Inkosi Albert Luthuli and King Edward VIII Hospital surgical intensive care units. Southern African J Crit Care (Online). 2015;31(1):16-8.

S98. Potgieter PD, Linton DM, Oliver S, Forder AA. Nosocomial infections in a respiratory intensive care unit. Crit Care Med. 1987;15:495-498.

S99. Boots RJ, Phillips GE, George N, Faoagali JL. Surveillance culture utility and safety using low-volume blind bronchoalveolar lavage in the diagnosis of ventilator-associated pneumonia. Respirology. 2008;13:87-96.

S100. Cade JF, McOwat E, Siganporia R, Keighley C, Presneill J, Sinickas V. Uncertain relevance of gastric colonization in the seriously ill. Intensive Care Med. 1992;18:210-7.
